# Luxation sous-talienne interne pure: à propos d’un cas

**DOI:** 10.11604/pamj.2017.27.123.12173

**Published:** 2017-06-15

**Authors:** Mustafa Nkaoui, Moncef Boufettal, Youness Sasbou, Mohammed Kharmaz, Mohamed El Ouadaghiri, Moulay Omar Lamrani, Ahmed El Bardouni, Mustapha Mahfoud, Mohamed Saleh Berrada

**Affiliations:** 1 Service de Chirurgie Orthopédique et de Traumatologie, CHU Ibn Sina, Université Mohammad V Souissi Rabat, Maroc

**Keywords:** Luxation sous-talienne interne, accident de sport, Internal subtalar dislocation, sport accident

## Abstract

La luxation sous talienne pure est une affection rare, les auteurs rapportent le cas d'un jeune patient ayant présenté suite à un accident de sport une luxation sous talienne interne pure, traité orthopédiquement avec un bon résultat fonctionnel.

## Abstract (en)

Pure subtalar dislocation is a rare condition. We here report the case of a young patient presenting with pure internal subtalar dislocation as a result of a sport accident. He underwent orthopedic therapy achieving a good functional outcome.

## Introduction

La luxation sous-talienne pure est le déplacement de l'ensemble calcanéo-pédieux au-dessous du talus maintenu dans la mortaise tibio-fibulaire, sans fracture associée. C´est une lésion rare, 1% de toutes les luxations observées en traumatologie.

## Patient et observation

**Figure 1:**
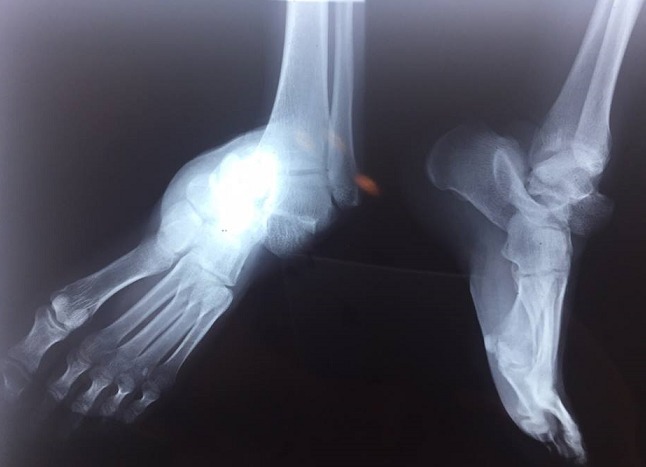
Aspect radiologique de la luxation sous-talienne pure interne en incidence face et profil

**Figure 2:**
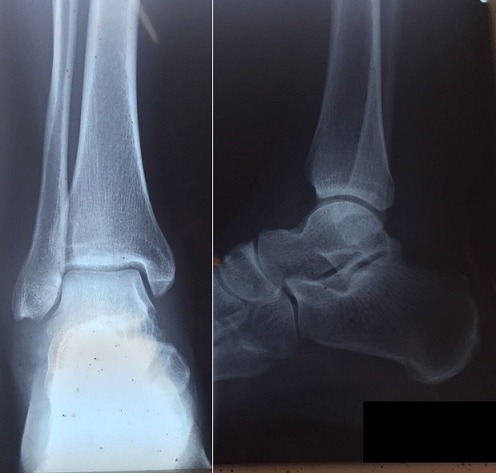
Radio de contrôle après réduction montrant une bonne congruence articulaire

C'est un sujet de sexe masculin, âgé de 28 ans, sans antécédents pathologiques notables. Il a été victime d'un traumatisme de la cheville droite suite à un accident de sport (lors d'un match de football) avec réception d'un saut en inversion et équinisme du pied droit. L'étude clinique avait objectivé une douleur avec impotence fonctionnelle du membre, une déformation de la région médio tarsienne avec œdème de la cheville, sans lésion cutanée ni vasculo-nerveuse. Les radiographies standards ont permis de poser le diagnostic d'une luxation sous talienne médiale pure (Figure 1). La réduction a été réalisée sous anesthésie générale par la manœuvre d'arrache botte, la cheville étant stable au testing et une radiographie de contrôle avait montré une bonne congruence articulaire (Figure 2). Ensuite, la cheville a été immobilisée dans une botte plâtrée pendant 6 semaines sans appui suivie d'une rééducation fonctionnelle. Le résultat fonctionnel était excellent avec un recul de 6 mois et la reprise sportive a été autorisée à 3 mois.

## Discussion

La luxation sous-talienne est une affection rare, très peu de cas ont été décrits dans la littérature souvent sous forme de cas isolé, la variété interne est la plus fréquente et c'est le fait que l'inversion est la principale position d'instabilité du pied qui explique cette grande fréquence [1, 2]. Le mécanisme de la luxation sous-talienne médiale est une inversion forcée avec pied bloqué au sol entrainant une rupture ligamentaire dans un ordre chronologique précis : c'est d'abord le ligament talo-naviculaire dorsal qui est lésé, puis les deux faisceaux du ligament interosseux ou ligament en haie et enfin le ligament péronéo-calcanéen [3]. La déformation clinique est évidente et le diagnostic est confirmé sur analyse des radiographies standards du pied et de la cheville surtout l'incidence face qui en montrant l'astragale en place dans la mortaise tibio-fibulaire alors que le pied est déplacé en interne [3]. Le traitement est la réduction en urgence sous anesthésie générale. Celle-ci se fait par la manœuvre de l'arrache-botte, le genou étant en flexion pour détendre le triceps sural. Une irréductibilité peut être le fait d'interpositions des tendons des muscles fibulaires, du ligament frondiforme, du muscle court extenseur des orteils ou d'un fragment osseux pour les luxations médiales. La réduction est habituellement stable et ne justifie, pour la luxation pure, aucune ostéosynthèse de principe [1] (botte plâtrée pendant 3 à 6 semaines sans appui). Le pronostic de ces lésions est relativement bon chez la plupart des auteurs sauf en cas d'ouverture cutanée ou de fracture associée [1, 4, 5]. Le risque de nécrose talienne est évalué à 4 % et celui d'arthrose sous-talienne à 31% [5].

## Conclusion

La luxation sous talienne interne pure est une affection traumatologique rare, son diagnostic est facile, le traitement consiste souvent en une réduction par manœuvre externe sous anesthésie générale, sauf en cas d'irréductibilité par incarcération ligamentaire ou une réduction chirurgicale s'impose. Ce sont des lésions de bon pronostic sauf dans les cas associés à une ouverture cutanée.

## Conflits d’intérêts

Les auteurs déclarent ne pas avoir de conflits d'intérêts en relation avec cet article.
